# Gemcitabine with either paclitaxel or vinorelbine *vs* paclitaxel or gemcitabine alone for elderly or unfit advanced non-small-cell lung cancer patients

**DOI:** 10.1038/sj.bjc.6602011

**Published:** 2004-07-13

**Authors:** P Comella, G Frasci, P Carnicelli, B Massidda, F Buzzi, G Filippelli, L Maiorino, M Guida, N Panza, S Mancarella, R Cioffi

**Affiliations:** 1 Division of Medical Oncology A, Department of Medicine, National Tumour Institute, Via M. Semmola, 80131 Naples, Italy; 2 Service of Medical Oncology, Da Procida Hospital, Via S. Calenda, 84100 Salerno, Italy; 3 Chair of Medical Oncology, University Medical School, Via SS 554, 09100 Cagliari, Italy; 4 Division of Medical Oncology, City Hospital, Via Joannuccio, 05100 Terni, Italy; 5 Service of Medical Oncology, City Hospital, Via Promintesta, 87027 Paola (Cosenza), Italy; 6 Service of Medical Oncology, San Gennaro Hospital, Via San Gennaro dei Poveri 5, 80131 Naples, Italy; 7 Division of Medical Oncology, Oncology Institute, Via Amendola 109, 70126 Bari, Italy; 8 Division of Medical Oncology, Cardarelli Hospital, Via Cardarelli, 80131 Naples, Italy; 9 Service of Medical Oncology, City Hospital, Via Taranto, 73021 Campi Salentino (Lecce), Italy; 10 Division of Pneumology, City Hospital, Via Tescione, 81100 Caserta, Italy

**Keywords:** elderly NSCLC patients, gemcitabine, paclitaxel, vinorelbine, doublet regimens

## Abstract

The aim of this study was to assess whether a combination of gemcitabine (GEM) with either paclitaxel (PTX) or vinorelbine (VNR) could be more effective than GEM or PTX alone in elderly or unfit advanced non-small-cell lung cancer (NSCLC) patients. A total of 264 NSCLC patients aged >70 years with ECOG performance status (PS)⩽2, or younger with PS=2, were randomly treated with: GEM 1200 mg m^−2^ on days 1, 8 and 15 every 28 days; PTX 100 mg m^−2^ on days 1, 8 and 15 every 28 days; GEM 1000 mg m^−2^ plus PTX 80 mg m^−2^ (GT) on days 1 and 8 every 21 days; GEM 1000 mg m^−2^ plus VNR 25 mg m^−2^ (GV) on days 1 and 8 every 21 days. In all arms, an intra-patients dose escalation was applied over the first three courses, provided that no toxicity of WHO grade ⩾2 had previously occurred. At present time, 217 (82%) patients had died. The median (months) and 1-year survival probability were 5.1 and 29% for GEM, 6.4 and 25% for PTX, 9.2 and 44% for GT, and 9.7 and 32% for GV. Multivariate analysis showed that PS⩽1 (hazard ratio (HR)=0.67; 95% CI 0.51–0.90), and doublet treatments (HR=0.76; 95% CI 0.59–0.99) were significantly associated with longer survival. Doublets produced no more toxicity than single agents. GT should be considered a reference regimen for elderly NSCLC patients with PS⩽1.

The standard approach for advanced non-small-cell lung cancer (NSCLC) is still a matter of debate. Indeed, there is a general consensus that cisplatin-based chemotherapy may produce a short but significant survival prolongation and improve the quality of life of patients in comparison with best supportive care alone (Non Small Cell Lung Cancer Collaborative Group, 1995; Cullen *et al*, 1999). Moreover, the Eastern Cooperative Oncology Group (ECOG) trial E1594 has recently shown that platinum doublets (cisplatin plus gemcitabine, cisplatin plus paclitaxel, carboplatin plus paclitaxel, or cisplatin plus docetaxel) have equivalent efficacy while differing in cost, toxicity and convenience (Schiller *et al*, 2002). However, there is still a diffuse concern about the tolerability of cisplatin in elderly and/or unfit patients (Earle *et al*, 2001; Bunn and Lilenbaum, 2003; Hennessy *et al*, 2003). As a matter of fact, the perceived rate of unacceptable toxicity among patients with poor performance status (PS) induced the discontinuation of their accrual in the above-mentioned ECOG E1594 study (Sweeney *et al*, 2001), and retrospective analyses on randomised trials have shown that PS 2 patients have no survival gain from chemotherapy (Billingham and Cullen, 2001; Soria *et al*, 2001).

As for elderly patients, two different approaches have recently emerged in literature. One of these has been to perform age-specific retrospective analyses on patients treated in randomised trials evaluating cisplatin-based doublets. Accordingly, several authors have recently reported neither substantial difference in survival, nor greater toxicity, for elderly patients treated with these regimens (Kelly *et al*, 2001; Rocha Lima *et al*, 2002; Hensing *et al*, 2003; Langer *et al*, 2003). However, these findings have the clear drawback of being extrapolated from studies carried out for different purposes.

A different approach for addressing this problem has been to explore in prospective trials the benefit of non-cisplatin-based treatments. The first one of these studies has been the ELVIS trial (Elderly Lung cancer Vinorelbine Italian Study Group, 1999), which showed an advantage in the median survival time (MST) (28 *vs* 21 weeks) for elderly patients receiving single agent vinorelbine as compared to those treated with supportive care alone. Single agent paclitaxel was also demonstrated to produce a 2-month longer MST (6.8 *vs* 4.8 months) in comparison with supportive care in a randomised trial with no upper limit of age for accrual (Ranson, 2000), and this drug appeared highly attractive for treating elderly patients, in view of the increasing evidence that a weekly schedule may improve its toxicity profile (Alberola *et al*, 2002). Furthermore, retrospective (Martin *et al*, 1997; Shepherd *et al*, 1997) and prospective studies (Altavilla *et al*, 2000; Anderson *et al*, 2000; Ricci *et al*, 2000) have also supported the use of gemcitabine in elderly NSCLC patients, given its good tolerability and activity regardless of age.

A further step ahead on this issue was made by the Southern Italy Cooperative Oncology Group (SICOG), which demonstrated that a combination of gemcitabine plus vinorelbine fared better than vinorelbine alone in elderly patients, obtaining a MST of 29 *vs* 18 weeks, and delaying the symptom and quality of life deterioration (Frasci *et al*, 2000). Moreover, this study highlighted that a high Charlson score (⩾3) was associated with the worst survival, suggesting that a comprehensive geriatric assessment, specifically looking at the type and number of the comorbidities, was mandatory in order to select the patients who may take advantage from a potentially active treatment (Frasci, 2002).

In the meantime, other investigators have explored the efficacy of original schedules of gemcitabine plus paclitaxel in NSCLC patients. For instance, Spanish investigators assessed a biweekly regimen with gemcitabine 2000 mg m^−2^ plus paclitaxel 150 mg m^−2^, obtaining a 32% response rate (RR), and a 9.9 months MST, in the absence of grade ⩾3 myelotoxicity (Isla *et al*, 2001). Hirsh *et al* (2003) administered gemcitabine 1000 mg m^−2^ plus paclitaxel 100 mg m^−2^, both on days 1 and 8 every 3 weeks, reporting a 55% RR and an MST of 9.8 months, with negligible myelosuppression. Therefore, the fractionated administration of paclitaxel and gemcitabine on the same days apparently increased the therapeutic index of the combination, supporting its use in elderly patients.

With these premises in mind, we decided to set up a new trial (SICOG 9909) restricted to elderly or unfit NSCLC patients with a Charlson score ⩽4. The primary aim of this trial was to assess whether the combination of gemcitabine plus either vinorelbine or paclitaxel could prolong the survival of patients in comparison with gemcitabine or paclitaxel alone. Secondary end points were time to treatment failure, response rate and toxicity. Furthermore, because at that time there was no agreement on the optimal dosage of these compounds for managing aged patients, and given the unpredictable tolerability of chemotherapy in elderly people, we used an individual dose optimisation (Frasci *et al*, 1998), starting treatment with the minimum active dose of each cytotoxic drug, applying a substantial dose reduction in the presence of haematologic toxicity as in our previous trial (Frasci *et al*, 2000), and assessing the feasibility of an intra-patient dose escalation only in the absence of a relevant toxicity on previous cycle.

## PATIENTS AND METHODS

### Patients selection

The main eligibility criteria for this study were: histologically or cytologically proven diagnosis of NSCLC; age >70 years associated with an ECOG PS ⩽2, or age ⩽70 years coupled with a PS 2; stage IIIB (not amenable to local treatment) or stage IV of disease; presence of measurable lesion(s); Charlson score no greater than 4; normal bone marrow reserve (neutrophil count ⩾2000 *μ*l^−1^, platelet count ⩾100 000 *μ*l^−1^, haemoglobin concentration ⩾10 g dl^−1^); adequate liver (bilirubin level <2 × the upper normal limit (UNL), AST and ALT <3 × UNL) and renal function (creatinine clearance ⩾60 ml min^−1^). Exclusion criteria were: presence of brain metastasis, uncontrolled metabolic or infectious diseases, presence of severe cardiac arrhythmia or heart failure, previous exposure to chemotherapy or radiotherapy, and previous diagnosis of malignant tumour within the last 5 years. The study protocol was approved by the Independent Ethical Committee of the National Tumour Institute of Naples, and written informed consent was required from each patient before registration.

Within a month before inclusion, all patients were submitted to a careful staging work-up, including history, physical examination, chest X-ray, ECG, computed tomography (CT) scan of thorax and upper abdomen, and bone radionuclide scan. Additional tests were performed only when clinically indicated. Within a week before registration, a full biochemistry profile, and a blood cell count with differential, were performed. Biochemistry was repeated at the start of each subsequent cycle, while blood cell count was performed weekly.

### Stratification, randomisation, and treatment

Patients were registered by fax at the coordinating centre. After verifying the eligibility criteria, patients were stratified according to stage (IIIB *vs* IV), PS (0–1 *vs* 2), and Charlson score (0–2 *vs* 3–4), and allocated using a computer-generated random list into one of four arms: gemcitabine (GEM), paclitaxel (PTX), gemcitabine plus paclitaxel (GT), or gemcitabine plus vinorelbine (GV). In all arms of the trial, at least three cycles were planned before the assessment of activity, and further treatment was administered up to a maximum of six cycles only if at least a disease control was demonstrated. In the absence of World Health Organization (WHO) grade ⩾2 toxicity on previous cycle, an intra-patient dose escalation over the first three cycles was planned in all arms of the trial, and the dosage reached in the third cycle was used thereafter.

In the GEM arm, the first cycle consisted of gemcitabine 1200 mg m^−2^ infused intravenously (i.v.) over 30 min on days 1, 8 and 15, recycling every 4 weeks. Gemcitabine could be increased to 1400 mg m^−2^ on the second cycle, and to 1600 mg m^−2^ on the third cycle. In the PTX arm, initial dose was 100 mg m^−2^ infused i.v. over 1 h on days 1, 8 and 15, recycling every 4 weeks. PTX could be increased to 120 mg m^−2^ on the second cycle, and to 140 mg m^−2^ on the third cycle. In the GT arm, paclitaxel 80 mg m^−2^ (over 1 h), followed by gemcitabine 1000 mg m^−2^ (over 30 min), was administered i.v. on days 1 and 8, recycling every 3 weeks. Gemcitabine could be increased to 1200 mg m^−2^ on the second cycle, while paclitaxel could reach 100 mg m^−2^ on the third cycle. In the GV arm, gemcitabine 1000 mg m^−2^ (in 30 min), and vinorelbine 25 mg m^−2^ (in 15 min) were given i.v. on days 1 and 8, recycling every 3 weeks; gemcitabine could be increased to 1200 mg m^−2^ on the second cycle, while vinorelbine could be increased to 30 mg m^−2^ on the third cycle.

In each arm of the trial, chemotherapy was administered in the presence of neutrophil count ⩾1500 dl^−1^, platelet count ⩾100 000 dl^−1^ and after complete recovery from previous nonhaematologic toxicity. In the presence of neutrophil count <1500 but ⩾1000 dl^−1^ and/or platelet count <100 000 but ⩾75 000 dl^−1^, a 50% dose reduction was applied for each drug. If lower values occurred on the initial day of each cycle, chemotherapy was postponed for a week, while doses were omitted if they did occur on day 8 or 15. Anti-emetic treatment and prevention of allergic reactions were provided according to standard guidelines. Supportive care was not defined in the study protocol, and it was left to investigator's choice. Treatment was discontinued in the case of documented tumour progression after three cycles, or earlier in the case of severe toxicity, deterioration of clinical condition, or withdrawal of patient's consent. Administration of palliative radiotherapy was left to the discretion of the attending physician. After discontinuation of study treatment, no further cytotoxic treatment was administered. Patients received symptomatic treatment, and were followed every month for the assessment of disease status and survival.

### Evaluation of toxicity and response

Toxicity was assessed after each cycle of treatment and scored according to WHO criteria (Miller *et al*, 1981). The worst degree of toxicity experienced throughout the treatment was recorded for each patient. Activity was defined by repeating a complete diagnostic work-up after three cycles, and at the end of therapy, and the best patient's response was recorded for the analysis. Responses were classified according to WHO criteria (Miller *et al*, 1981). Early treatment discontinuation or death for any cause were considered as treatment failures. Duration of complete response was measured from the date it was first documented, while duration of partial response was calculated from the date of randomisation to the date of progression or death, whichever occurred first.

### Evaluation of survival

Survival of patients was calculated from the date they were randomised to the date of death or last follow-up. All patients treated as allocated as well as patients not treated according to random assignment were included in this analysis according to the intent-to-treat principle. Failure-free survival was calculated for each patient from the date of randomisation to the date of progression or failure.

### Statistical analysis and sample size

Proportions were calculated with 95% exact confidence interval (95% CI) and compared using the Fisher's exact test. Actuarial survival and failure-free survival curves were generated using the Kaplan and Meier method (Kaplan and Meier, 1958) and compared using a two-sided log-rank test (Mantel, 1996). All statistical analyses were performed using the software SPSS software package, version 8.0 (SPSS Inc., Chicago, IL, USA).

The sample size was defined assuming from previous experience that both single-agent treatments could obtain a MST of 5 months. To detect an improvement to 7.5 months with a doublet regimen, a total of 257 events were required, giving a 90% power to obtain a *P*-value <0.05 at the log-rank test. Therefore, a total sample size of 130 patients per arm was planned. The enrolment started in May 1999, and we anticipated to complete the case accrual in about 4 years. However, in March 2003 we decided to close the recruitment, in consideration of the slower than anticipated accrual, and on the basis of some ethical concern raised by a recently published trial in elderly patients, in which no survival benefit derived for the combination of gemcitabine plus vinorelbine over gemcitabine alone (Gridelli *et al*, 2003). Participating investigators were required to perform an *ad hoc* analysis of survival for patients enrolled until that date, allowing for a 6-month minimum follow-up after the last patient had been recruited. A multivariate analysis with a backward selection procedure (Cox, 1972) was also applied to evaluate the best factors independently affecting survival, including as discrete covariates: age of patients (more or less than 70 years), performance status (0–1 or 2), previous weight loss (yes or not), Charlson score (0–2 or 3–4), histologic subtype (squamous carcinoma, adenocarcinoma, or other subtypes), stage of disease (IIIB or IV) and treatment (single agent or doublet).

## RESULTS

### Patient characteristics

**Figure 1:**
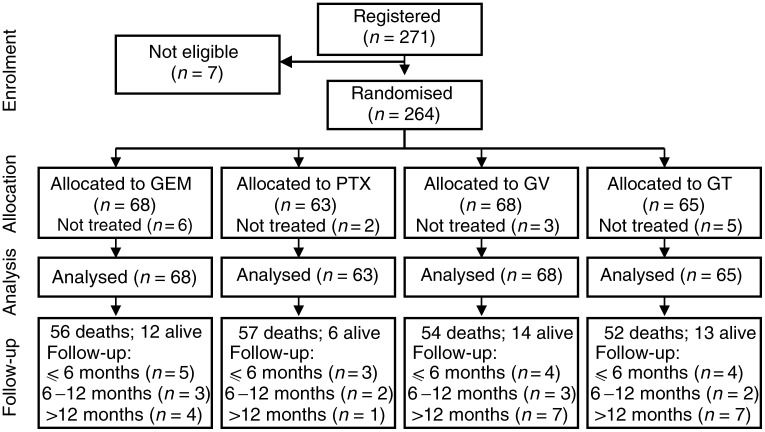
Consolidation of Standards for Reporting Trials (CONSORT) flow chart of the study.

From May 1999 to March 2003, 271 patients were registered into this study. However, seven patients were not randomised because of wrong histology (one patient), or no available baseline information about requirements of eligibility (six patients). In all, 264 patients were randomly allocated to one of four arms (Figure 1

). A total of 16 patients did not receive treatment as allocated, because of withdrawal of patient's consent (five cases), or because of attending physician's decision (11 cases): these patients were not considered in the analysis of activity and toxicity, but were included in the survival analysis.

As detailed in Table 1

**Table 1:** Characteristics of patients enrolled in the SICOG trial 9909 according to the arm of treatment

	**GEM**		**PTX**		**GV**		**GT**	
**Characteristics**	**No.**	**(%)**	**No.**	**(%)**	**No.**	**(%)**	**No.**	**(%)**
Number of patients	68	(100)	63	(100)	68	(100)	65	(100)
Males	57	(84)	57	(90)	63	(93)	59	(91)
Females	11	(16)	6	(10)	5	(7)	6	(9)
Median age (range)	75	(49–86)	72	(50–81)	72	(42–82)	73	(53–83)
Age⩽70 years	9	(13)	13	(21)	13	(19)	9	(14)
Weigh loss >5%	23	(34)	31	(49)	18	(27)	19	(29)
PS 0–1	49	(72)	41	(65)	47	(69)	50	(77)
PS 2	19	(28)	22	(35)	21	(31)	15	(23)
Stage III	24	(35)	16	(25)	28	(41)	25	(38)
Stage IV	44	(65)	47	(75)	40	(59)	40	(62)
2+ metastatic sites	13	(30)	10	(21)	10	(26)	8	(21)
Squamous carcinoma	27	(40)	33	(52)	34	(50)	33	(51)
Adenocarcinoma	24	(35)	18	(29)	13	(19)	16	(25)
Large-cell carcinoma	3	(4)	4	(6)	10	(15)	3	(4)
Unclassified	14	(21)	8	(13)	11	(16)	13	(20)

GEM=gemcitabine, PTX=paclitaxel, GV=gemcitabine plus vinorelbine, GT=gemcitabine plus paclitaxel.

, most patients (89%) were males. In all, 220 (83%) patients were older than 70 years. Among these, 93 (35%) patients were aged ⩾75 years, and 14 (5%) were aged ⩾80 years. However, 44 (17%) patients aged ⩽70 years were also enrolled because of their poor PS. Regardless of age, 77 (29%) patients had an ECOG PS 2. Squamous cell carcinoma accounted for 48% of all diagnoses, followed by adenocarcinoma (27%). In total, 98 (37%) patients were classified in stage IIIB, and 166 (63%) patients were in stage IV; 41 (25%) of these patients had more than one metastatic site of disease. A recent weight loss was registered in 91 (34%) patients. In 87 (33%) patients no associated diseases were recorded, while 161 (61%) patients had a Charlson score 1 or 2, and 16 (6%) patients had a score ⩾3 (Table 2

**Table 2:** Type of comorbidities, and Charlson score, according to arms of treatment

	**GEM**		**PTX**		**GV**		**GT**	
**Associated diseases**	**No.**	**%**	**No.**	**%**	**No.**	**%**	**No.**	**%**
Myocardial infarction	2	3	3	5	1	4	3	5
Chronic cardiac failure	5	7	1	1	2	3	2	3
Peripheral vascular	5	7	8	12	3	4	8	12
Cerebral vascular	7	10	3	5	4	6	4	6
Dementia	1	1	0	0	0	0	0	0
Chronic bronchopneumonia	20	29	21	32	19	28	19	29
Connectivitis	1	1	0	0	1	4	2	3
Peptic ulcer	2	3	3	5	2	3	5	8
Chronic hepatitis	1	1	2	3	2	3	7	11
Diabetes	3	4	6	9	4	6	5	8
None	23	34	20	32	21	31	23	35
								
*Charlson score*								
0	23	34	20	32	21	31	23	35
1–2	42	62	36	57	43	63	40	62
3–4	3	4	7	11	4	6	2	3

GEM=gemcitabine, PTX=paclitaxel, GV=gemcitabine plus vinorelbine, GT=gemcitabine plus paclitaxel.

). All pre-treatment characteristics resulted well balanced across the four arms of the trials.

### Survival and failure-free survival

**Figure 2:**
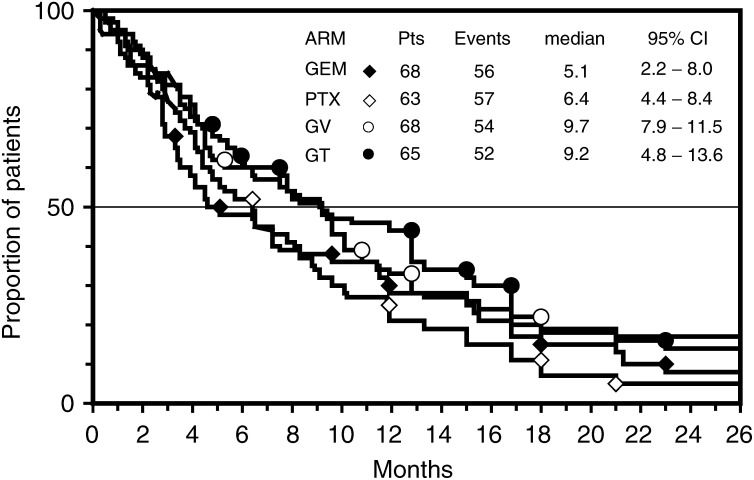
Actuarial survival curves of patients according to the four arms of the trial: GEM arm (black diamond), PTX arm (white diamond), GV arm (open circle), GT arm (close circle). The differences did not reach a significant *P*-value.

Survival was the main end point of this study, and all 264 eligible patients were included in this analysis according to intent-to-treat principle. By September 30, 2003, the median potential follow-up for the whole series was 33 (range, 6–52) months, with no difference between arms of treatment. At that time, 219 (83%) patients had died, and MST for the whole series was 7.5 (95% CI, 5.9–9.1) months. Early death (within 60 days from randomisation) occurred in 14 (21%) patients of the GEM arm, in eight (13%) patients of the PTX arm, in nine (14%) patients of the GT arm and in nine (13%) patients of the GV arm. Both doublets produced a longer survival than single agents (Figure 2

). Indeed, MST and 1-year survival rate (1-year SR) were 9.2 months and 44% for patients treated with GT, 9.7 months and 32% for patients treated with GV, 6.4 months and 25% for patients treated with PTX, and 5.1 and 29% for patients treated with GEM. Difference between GT and PTX approached the significance level (*P* value=0.051), while difference between GV and GEM was not significant.

**Figure 3:**
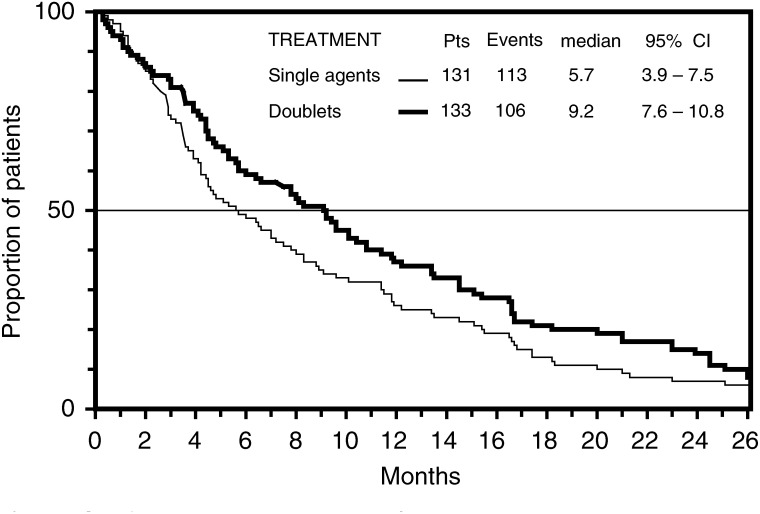
Actuarial survival curves of patients treated with either single agent (thin line) or with either doublet (thick line). The difference was significant at the log-rank test (*P*=0.028).

The pooled comparison of survival of patients treated with either GEM or PTX *vs* patients treated with either GV or GT showed a significant difference in favour of doublet regimens (Figure 3

). Indeed, the MST and 1-year SR were 5.7 (95% CI, 3.9–7.5) months and 28% for single-agent treatments, and 9.2 (95% CI, 7.6–10.8) months and 39% for the combinations (*P*-value=0.028). The multivariate analysis showed that only PS and treatment independently affected the survival of patients. Indeed, the hazard ratio of death was 0.67 (95% CI, 0.51–0.90) for patients with performance status 0 or 1 as compared to patients with poorer performance status (*P*-value=0.0068), and it was 0.76 (95% CI, 0.59–0.99) for patients treated with a doublet compared to patients treated with a single agent (*P*-value=0.0486).

**Figure 4:**
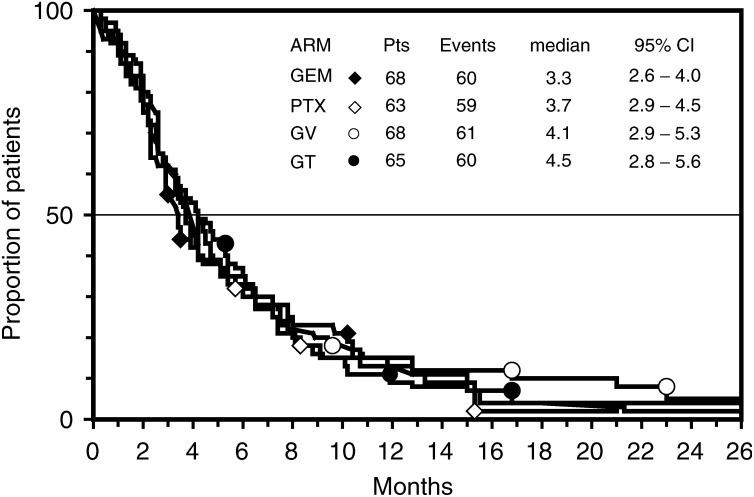
Actuarial failure-free survival curves of patients according to the four arms of the trial: GEM arm (black diamond), PTX arm (white diamond), GV arm (white circle), or GT arm (black circle). The differences did not reach a significant *P*-value.

The failure-free survival curves are plotted in Figure 4

. Also in this case, both doublets were associated with a longer failure-free survival than each single-agent regimen. The median failure-free interval (in months) was 4. 5 for patients treated with GT, and 4.1 for patients treated with GV, while it was 3.7 for PTX, and 3.3 for GEM. However, these differences did not reach a significant level.

### Administered treatment and dose escalation

In all arms, median number of administered cycles was three (range, 1–6) per patient. Early treatment discontinuation, due to rapid deterioration of clinical conditions, occurred in similar proportions of patients in all arms of the study (Table 3

**Table 3:** Administered cycles according to arms of treatment

	**GEM**		**PTX**		**GV**		**GT**	
**Treatment**	**No.**	**%**	**No.**	**%**	**No.**	**%**	**No.**	**%**
Not treated patients	6	9	2	3	3	4	5	8
Treated patients	62	91	61	97	66	96	60	92
Total cycles	176		175		233		219	
								
*Median cycles/patient*	3		3		3		3	
⩾2 cycles	50	81	47	77	53	80	51	85
⩾3 cycles	36	58	37	61	52	79	48	80
⩾4 cycles	13	21	13	21	24	36	23	38
⩾5 cycles	9	15	10	16	22	33	20	33
⩾6 cycles	7	11	7	11	15	23	14	23

GEM=gemcitabine, PTX=paclitaxel, GV=gemcitabine plus vinorelbine, GT=gemcitabine plus paclitaxel.

). Early withdrawal was significantly associated with baseline PS of patients. Indeed, 35 (45%) of 77 patients with PS 2 received less than three courses as compared to 42 (24%) of 172 patients with PS 0 or 1 (*P*-value=0.0009).

In GEM arm, a dose escalation was performed on the second cycle in 24 (48%) patients, and the mean administered dose was 1286 mg m^−2^. On the third cycle, gemcitabine was further escalated in 14 (39%) patients, and the mean dose was 1371 mg m^−2^. Dose reductions or omissions occurred in 37% of patients on the first cycle, in 32% of patients on the second cycle and in 39% of patients on the third cycle. First PTX dose escalation was performed in 26 (55%) patients, and the mean dose on the second cycle was 111 mg m^−2^. On the third cycle, an escalation was applied in 16 (43%) patients, and the mean dose given was 119 mg m^−2^. Reduction or omissions were required in 18% of patients on the first cycle, in 15% of patients on the second cycle and in 19% of patients on the third cycle. In the GT arm, gemcitabine was increased in 26 (51%) patients, and its mean dose on the second cycle was 1094 mg m^−2^, while paclitaxel was increased in 24 (50%) patients, and its mean dose on the third cycle was 89 mg m^−2^. Dose reductions occurred in 23% of patients on the first cycle, in 22% of patients on the second cycle and in 13% of patients on the third cycle. In the GV arm, gemcitabine was escalated in 21 (40%) patients, and its mean dose on the second cycle was 1080 mg m^−2^, while vinorelbine was increased in 12 (24%) patients on the third cycle, and its mean dose was 26 mg m^−2^. Dose omissions occurred in 15% of patients on the first cycle, in 11% of patients on the second cycle and in 10% of patients on the third cycle.

### Activity

In all, 11 patients showed a partial response in GEM arm, for an RR of 18% (95% CI, 9–30%); eight patients obtained a partial response in PTX arm, giving an RR of 13% (95% CI, 6–24%); 19 partial responses were achieved with GT regimen, giving an RR of 32% (95% CI, 20–45%); one complete plus 14 partial responses were registered among patients treated with GV, giving an RR of 23% (95% CI, 13–35%). RRs in metastatic patients were 18, 15, 25 and 25%, respectively, for the GEM, PTX, GT and GV regimens. Patients with better PS had a higher RR than patients with poor PS: RR was 17 *vs* 15% in the GEM arm, 17 *vs* 5% in PTX arm, 28% *vs* no response in the GT arm and 38 *vs* 9.5% in the GV arm. Conversely, no substantial difference of activity was apparent among patients age ⩾75 years: RR was 15 and 5% in the GEM and PTX arms, respectively, while it resulted 29% in both combination arms.

The overall activity of GT resulted significantly greater than that of PTX (*P*-value=0.013), while the comparison of GV *vs* either single agent did not reach a level of significance. Noteworthy, the proportion of patients achieving a control of tumour growth (major response or stabilisation) was 37% in the GEM arm, 34% in the PTX arm, 60% in the GT arm and 53% in the GV arm. In this respect, both doublets resulted significantly more active than single agents.

Considering the patients treated with at least three cycles, a higher proportion of responses occurred among patients able to tolerate a dose escalation of both drugs in the GT or GV arm, as opposed to those in whom escalation was limited to gemcitabine, or not feasible at all: responders were 12 out of 24 (50%) patients in the GT arm, and seven out of 12 (58%) patients in the GV arm, as opposed to seven out of 24 (29%) and eight out of 40 (20%) patients, respectively. Conversely, no substantial difference in RR according to drug escalation was observed in GEM or PTX arm. Responders were four out of 14 (29%) and four out of 16 (25%) patients, respectively, receiving a full dose escalation over three cycles, and seven out of 22 (32%) and four out of 21 (19%) patients, respectively, with partial or no dose escalation.

Duration of major responses ranged from 4.6 to 15.0 months, with no substantial differences across the four arms of the study (Table 4

**Table 4:** Summary of activity according to arms of treatment

**Outcome**	**GEM**	**PTX**	**GV**	**GT**
*Response rate*	18	13	23	32
95% CI	9–30	6–24	13–35	20–45
				
*Disease control rate*	37	34	53	60
95% CI	25–50	23–48	40–65	45–71
				
*Duration of responses*				
Median (months)	6.8	6.6	7.6	6.5
Range	4.6–15	4.2–9.0	5.0–10.2	5.8–8.2
				
*Survival*				
Median (months)	5.1	6.4	9.7	9.2
95% CI	2.2–8.0	4.4–8.4	7.9–11.5	4.8–13.6
				
*1-year survival probability*	29	25	32	44
95% CI	17–41	13–37	20–44	32–56
				
*2-year survival probability*	8	8	14	11
95% CI	1–16	1–16	4–24	1–21
				
*Failure-free survival*				
Median (months)	3.3	3.7	4.1	4.5
95% CI	2.6–4.0	2.9–4.5	2.9–5.3	2.8–5.6
6-month probability	33	31	33	36

GEM=gemcitabine, PTX=paclitaxel, GV=gemcitabine plus vinorelbine, GT=gemcitabine plus paclitaxel.

).

### Toxicity

No toxic deaths were registered in this trial. Severe neutropenia occasionally occurred, and few patients in each arm experienced febrile neutropenia (Table 5

**Table 5:** Acute haematologic toxicity according to arm of treatment

		**GEM (59)**		**PTX (60)**		**GV (61)**		**GT (57)**	
**WHO toxicity**	**Arm (patients)**	**No.**	**%**	**No.**	**%**	**No.**	**%**	**No.**	**%**
Neutropenia	G1	10	17	7	12	5	8	9	16
	G2	3	5	8	13	11	18	9	16
	G3	7	12	3	5	5	8	3	5
	G4	3	5	2	3	4	6	2	3
									
Febrile neutropenia		2	3	2	3	3	5	1	2
									
Thrombocytopenia	G1	4	7	2	3	7	11	7	12
	G2	6	10	0	0	5	8	6	11
	G3	3	5	0	0	1	2	2	3
	G4	3	5	0	0	1	2	2	3
									
Anaemia	G1	7	12	5	8	7	11	6	11
	G2	2	3	1	2	6	10	7	12
	G3	5	8	1	2	3	5	2	2
	G4	1	2	0	0	0	0	0	0

GEM=gemcitabine, PTX=paclitaxel, GV=gemcitabine plus vinorelbine, GT=gemcitabine plus paclitaxel.

). Severe thrombocytopenia occurred in six patients (three cases of grade 4) of the GEM arm, in two patients (one case of grade 4) of the GV arm, and in four patients (two cases of grade 4) of the GT arm. However, no episodes of bleeding occurred, nor platelet transfusions were required. Anaemia of any grade affected a similar proportion of patients in GEM (25%), GT (25%) and GV (26%) arms, while it was very rare in the PTX arm (12% of patients). Furthermore, we would remark that, among patients treated with GEM alone, severe anaemia occurred more frequently in patients aged ⩾75 years (13%) than in younger ones (7%), while no difference according to age grouping was seen in the other arms of the study. Packed red cell transfusions were given during treatment to four patients of the GEM arm, and to one patient each of the GV and GT arms.

Nonhaematologic toxicity was quite mild, and few episodes of grade ⩾3 toxicity of any type were reported (Table 6

**Table 6:** Acute nonhaematologic toxicity according to the arm of treatment

		**GEM (59)**		**PTX (59)**		**GV (61)**		**GT (57)**	
**WHO toxicity**	**Arm (Patients)**	**No.**	**%**	**No.**	**%**	**No.**	**%**	**No.**	**%**
Vomiting	G1–2	9	15	14	24	14	23	10	18
	G3	2	3	0	0	2	3	2	4
Diarrhoea	G1–2	2	4	5	8	4	7	6	11
	G3–4	0	0	1	2	0	0	1	2
Stomatitis	G1–2	2	3	7	12	5	8	5	9
	G3–4	1	2	0	0	1	3	0	0
Neurologic	G1–2	1	2	4	7	4	7	6	11
	G3–4	1	2	0	0	1	3	1	2
Renal	G1–2	2	4	0	0	2	3	2	4
	G3	1	2	0	0	0	0	0	0
Hepatic	G1–2	1	2	0	0	4	7	3	5
	G3	0	0	0	0	0	0	1	2
Alopecia	G1–2	8	14	12	20	17	28	11	19
	G3	0	0	4	7	0	0	7	12
Allergic	G1–2	3	5	3	5	4	7	3	5
Constipation	G1–2	2	4	2	3	4	7	1	3

GEM=gemcitabine, PTX=paclitaxel, GV=gemcitabine plus vinorelbine, GT=gemcitabine plus paclitaxel.

). Mild–moderate stomatitis occurred in seven (12%) patients treated in the PTX arm, and in five (9%) patients treated in the GT arm. Neurologic toxicity of any grade was seen in five patients (10%) treated with GV, in seven patients (13%) treated with GT, in four patients (7%) patients treated with PTX and in two patients (4%) treated with GEM. However, it should be remembered that twice as many patients in GT or GV arm received six cycles as compared to the GEM or PTX arm.

## DISCUSSION

The aim of this study was to ascertain whether a regimen including gemcitabine plus either vinorelbine or paclitaxel could prolong the survival of elderly patients with locally advanced or metastatic NSCLC patients in comparison with a single-agent treatment with gemcitabine or paclitaxel. We selected these doublets on the ground of their exciting activity demonstrated in our own previous experience (Frasci *et al*, 2000; Lorusso *et al*, 2000; Frasci, 2002), as well as in other studies (Isla *et al*, 2001; Hirsh *et al*, 2003), while paclitaxel and gemcitabine were identified as single agents to be challenged on the basis of several retrospective and prospective reports about their efficacy and tolerability in NSCLC patients.

The results reported here showed that both doublets produced a longer survival than either single agent. Failure-free survival analysis also showed an advantage for patients treated with either GV or GT in comparison with patients treated with either gemcitabine or paclitaxel. The higher activity of doublets in comparison with single agents was confirmed by the greater response and disease control rates. However, differences in survival did not reach a significance level. As we had decided to prematurely close the case accrual before the planned sample size of patients had been reached, we may speculate that these findings may be due to the low power of the test performed on a small patient population. For this reason, and given the similar outcome of patients treated with either single agent, as well as of patients treated with either doublet, we performed a pooled analysis, which showed a significant difference in favour of doublets. Furthermore, multivariate analysis of factors independently affecting the probability of survival revealed that only a poor performance status and a single-agent treatment were significantly associated with a shorter outcome.

It is worth noting that the actual MST of patients treated with either single agent was very close to that assumed in the design of the study, while patients treated with doublets fared even better than hypothesised. However, we have to underline that these results were obtained in a carefully selected patient population, as reflected by the lower than anticipated accrual rate. Indeed, while the proportion of patients with poor PS was similar in this (29%) and in our previous trial (24%) (Frasci *et al*, 2000), patients with brain metastases were excluded from the present trial. In addition, the present series was affected by less comorbidities. The adverse impact of the number of associated diseases, summarised by the Charlson score, on survival of elderly NSCLC patients was already demonstrated in our previous study (Frasci, 2002), and it has been confirmed in the present trial. Indeed, MST for patients with a Charlson score ⩾3 was 16 weeks in our previous study, and 4.0 months in the present trial, while it was 23 weeks and 7.6 months, respectively, for patients with a lower score. However, patients with a Charlson score ⩾3 were 18% in the previous trial and only 6% in the present series. These differences could partially explain the better than anticipated outcome observed in patients treated with doublets. Indeed, it may be argued that patients with a favourable prognosis could have benefited more from an active treatment than patients for whom life expectancy was already heavily compromised by their comorbidities.

In contrast to the present study, the Multicenter Italian Lung cancer in Elderly Study (MILES) showed no survival benefit from gemcitabine plus vinorelbine (MTS, 30 weeks) *vs* either single agent (MST, 28 and 36 weeks, respectively) (Gridelli *et al*, 2003). However, 20% of patients entered in the MILES trial were affected by three, and 25% of patients by four or more associated diseases. We wonder whether the greater toxicity, without any survival advantage, elicited in the MILES trial by the combination as compared with each of the components may be explained by this observation. Another possible explanation for the apparent discrepancy between MILES and SICOG 9909 studies may rely on the slightly lower dosages used in that trial for the combination (vinorelbine 25 mg m^−2^ plus gemcitabine 1000 mg m^−2^) than for each single-agent arm (vinorelbine 30 mg m^−2^ and gemcitabine 1200 mg m^−2^). Although a dose–response relationship is unproven in NSCLC, it is likely that at least an additive effect may occur when full doses of both drugs are combined. As a matter of fact, in both doublet arms of our study, but not in the single-agent arms, RR was greater among patients who could tolerate a dose escalation over the first three cycles. Noteworthy, we were able to increase both gemcitabine and vinorelbine in about a quarter of patients treated with GV. On the other hand, vinorelbine in the MILES trial produced an unexpectedly long-lasting MST, not only longer than the combination, but also greater than that obtained in the previous ELVIS study (Elderly Lung cancer Vinorelbine Italian Study Group, 1999), and even superior to those (ranging from 30 to 32 weeks) reported with this drug in three large randomised trials unrestricted to elderly patients (Depierre *et al*, 1994; Le Chevalier *et al*, 1994; Crawford *et al*, 1996).

As for the single agents of our trial, we would stress that MST (5.1 months) and 1-year SR (30%) obtained in the GEM arm were comparable to those achieved by gemcitabine either in the series without an upper age limit for inclusion, or in trials specifically designed for elderly patients. Indeed, an MST of 5.7 months and a 25% 1-year SR were reported for patients treated with GEM in a randomised study comparing this drug to the best supportive care (Anderson *et al*, 2000). In a phase II study restricted to patients aged more than 70 years, an MST of 6.7 months was achieved with GEM alone (Ricci *et al*, 2000). Moreover, Quoix (Quoix *et al*, 2003) recently explored in elderly patients the activity of two different schedules of gemcitabine (either 1000 mg m^−2^ for 3 consecutive weeks every 4 weeks, or 1125 mg m^−2^ for 2 consecutive weeks every 3 weeks), reporting a MST of 5.1 and 6.8 months, respectively. In the MILES trial, gemcitabine 1200 mg m^−2^ on days 1 and 8 every 3 weeks obtained a MST of 28 weeks, and a 28% 1-year SR (Gridelli *et al*, 2003).

As for the PTX arm, MST (6.4 months) and 1-year SR (24%) achieved in our study were comparable to those (6.8 months, and 35%, respectively) reported in a phase III trial comparing this drug with supportive care alone (Ranson *et al*, 2000). Moreover, in a large randomised study evaluating the addition of carboplatin to PTX in NSCLC patients, the single-agent PTX treatment yielded in the subset of elderly patients a MST of 5.0 months, with 1-year SR of 31% (Bunn and Lilenbaum, 2003).

One of the secondary end points of this study was to assess whether there was a substantial difference in safety between the regimens utilised. In this regard, we can state that no additional toxicity derived from the combination of two drugs in comparison with a single agent. Among doublets, similar proportions of patients treated with either GV or GT were affected by side effects of any grade, and few episodes of grade 4 neutropenia and/or febrile neutropenia, as well as of severe nonhaematologic toxicity, occurred in both arms. These findings were likely a consequence of the tailored approach we have used, applying very cautious rules for dose reduction/omission in the presence of haematologic toxicity, escalating the cytotoxic drugs only in the absence of relevant toxicity on previous cycle, and discontinuing any chemotherapy in patients showing no clear benefit from such treatment. This prudential approach has been adopted in consideration of the unpredictable occurrence in elderly subjects of side effects from cytotoxic drugs also in the presence of apparently normal organ functions (Lichtman *et al*, 1999; Aapro *et al*, 2000). As a matter of fact, the occurrence of severe bone marrow toxicity was even lower in the present trial than in our previous study (Frasci *et al*, 2000), in which full doses of gemcitabine (1200 mg m^−2^) plus vinorelbine (30 mg m^−2^) were administered from the beginning, while dose-reduction rules during treatment were exactly the same. Besides the PS of patients, we were unable to identify other pre-treatment features significantly affecting the treatment compliance in this study. Since the proportion of patients with poor PS did not differ in this and in our previous trial, we may argue that the pragmatic adaptation of treatment intensity represents a good way to improve the tolerability of cytotoxic drugs in elderly people.

In conclusion, this trial showed that survival of elderly advanced NSCLC patients, carefully selected on the basis of their Charlson score, may be prolonged using a non-cisplatin-based doublet, at the price of acceptable side effects. The significantly greater efficacy of doublets over a single agent was confirmed in the multivariate analysis. GT combination was associated with the best therapeutic index, and should be considered a reference regimen for these patients. However, a poor performance status was independently associated with a worse survival regardless of treatment employed. Therefore, future trials exploring new regimens should be restricted to patients with an ECOG PS ⩽1.
